# Microcrystalline Cellulose for Delivery of Recombinant Protein-Based Antigen against Erysipelas in Mice

**DOI:** 10.1155/2018/7670505

**Published:** 2018-06-11

**Authors:** Wooyoung Jeon, Yeu-Chun Kim, Minhee Hong, Sanoj Rejinold, Kyoungmoon Park, Injoong Yoon, Sungsik Yoo, Hongweon Lee, Jungoh Ahn

**Affiliations:** ^1^Biotechnology Process Engineering Center, KRIBB, Cheongju 363-883, Republic of Korea; ^2^Department of Chemical and Biomolecular Engineering, KAIST, Daejeon 305-701, Republic of Korea; ^3^Biological and Chemical Engineering, Hongik University, Sejong 339-701, Republic of Korea; ^4^Choong-Ang Vaccine Laboratory, Daejeon 305-348, Republic of Korea; ^5^University of Science and Technology (UST), 217 Gajeong-ro, Yuseong-gu, Daejeon 305-350, Republic of Korea

## Abstract

The study describes the development of a vaccine using microcrystalline cellulose (Avicel PH-101) as a delivery carrier of recombinant protein-based antigen against erysipelas. Recombinant SpaA, surface protective protein, from a gram-positive pathogen* Erysipelothrix rhusiopathiae* was fused to a cellulose-binding domain (CBD) from* Trichoderma harzianum* endoglucanase II through a S3N10 peptide. The fusion protein (CBD-SpaA) was expressed in* Escherichia coli* and was subsequently bound to Avicel PH-101. The antigenicity of CBD-SpaA bound to the Avicel was evaluated by enzyme-linked immunosorbent (ELISA) and confocal laser scanning microscope (CLSM) assays. For the examination of its immunogenicity, groups of mice were immunized with different constructs (soluble CBD-SpaA, Avicel coated with CBD-SpaA, whole bacterin of* E. rhusiopathiae* (positive control), and PBS (negative control)). In two weeks after immunization, mice were challenged with 1x10^7^ CFU of* E. rhusiopathiae* and Avicel coated with CBD-SpaA induced protective immunity in mice. In conclusion, this study demonstrates the feasibility of microcrystalline cellulose as the delivery system of recombinant protein subunit vaccine against* E. rhusiopathiae *infection in mice.

## 1. Introduction

Vaccines are the therapeutic formulations given to patients to elicit immune responses entailing antibody production (humoral) or cell-mediated responses that will eventually fight variety of malignancies [1]. There have been many approaches for vaccine delivery via vaccinations, which are considered the most efficient prophylactic method against various infectious diseases. Vaccine delivery systems (i.e., micro- or nanoparticles, liposomes, and virosomes) have been investigated for improving vaccine efficacy [2–5]. In addition, various vaccine types have been developed to overcome the disadvantages of conventional vaccines. Among vaccines, recombinant protein antigens, or their fragments, have been used and considered as novel vaccine candidates. Development of such kinds of vaccines avoids the safety issues with attenuating virus or cell cultures when making conventional viral vaccines [6, 7]. However, some recombinant protein antigens have fatal handicaps (low stability and immunogenicity) caused by lack of key elements that can stimulate immune response [8]. Use of adjuvants, along with recombinant protein vaccines, has been suggested for improved immunogenicity [9]. Different strategies have been extensively explored in order to improve the immunogenicity by protecting antigens through immobilization on inorganic or organic matrices [10].

Cellulose is a primary component of plant cell walls and a linear polymer of glucose residues. It is naturally resistant to biological degradation owing to its insolubility, rigidity, and tendency to pack together to form long crystals. It is chemically inert, pharmaceutically safe, and inexpensive, making it an effective immunosorbent, or carrier material, for protein purification and immobilization [11]. Cellulose-binding domain (CBD) refers to protein modules that bind firmly to various types of cellulose. The strong attraction between cellulose and CBD has applications in many areas, especially for immunology [12]. In previous studies, cellulose beads (Sigmacell 20® or Orbicell™ cellulose particles) attached to CBD-fused recombinant protein were used for parenteral vaccination for goldfish [13], and the immunogenic properties of CBD in its free form were compared with the CBD-cellulose complex, using cytokine production as an immunological indicator. Small Orbicell beads (1–10 *μ*M) induced antibody levels that were equal to the titers produced by the adjuvanted protein and bacterin formulae compared to the larger Sigmacell particles (10–20 *μ*M). Similarly, Lunin et al. (2009) studied the immobilization of CBD on the cellulose sorbent which enhances the synthesis of specific antibodies and found that cellulose immunosorbent is not immunotolerant and could induce cytokine production involved in the regulation of humoral immune response [8].

Erysipelas is a very serious swine disease causing tremendous economic loss.* E. rhusiopathiae*, causative agent of erysipelas, is a gram-positive, rod shaped, non-spore-forming, non-motile, and bacterial pathogen [14]. Although live-attenuated or recombinant vaccines are currently administered to control this disease, their effectiveness is not consistent. Even the mechanisms of pathogenicity have not yet been elucidated [15–18]. Therefore, it is necessary to have a more advanced and effective system of vaccination against erysipelas.

In this study, we developed Avicel (cellulose microcrystalline) correlated with antigen and investigated its characteristics. In addition, antigen-immobilized Avicel vaccine was subcutaneously injected into mice to analyze the actual immunogenicity of our newly developed antigen-immobilized Avicel recombinant protein vaccine. Overall, the study highlights the development of an efficient recombinant protein vaccine-immunosorbent system to protect swine against* E. rhusiopathiae* infection.

## 2. Materials and Methods

### 2.1. Bacterial Strains and Culture Conditions

The* E*.* rhusiopathiae* serotype 15 strain was isolated from a diseased piglet in Korea. For it,* E. coli* DH5*α* [F^−^, *ϕ*80d*lac*ZΔM15,* end*A1* hsd*R17 (rK^−^, mK^+^),* sup*E44,* thi-*1*, λ*^−^*, rec*A1*, gyr*A96] was used as a host strain for cloning and plasmid maintenance. The* E. coli* BL21 host strain [F^−^*, hsd*S*, gal, omp*T, rB^−^, mB^−^] (Novagen, USA), harboring a lambda derivative,* DE3*, was used for gene expression.

### 2.2. Cloning and Recombinant Plasmid Construction

The gene coding CBD of* Trichoderma harzianum *endoglucanase II, with artificial S_3_N_10_ linker, was amplified from pEKPM-EcGAD-Lk-H6 (Ahn, 2004; Hyemin Park, 2012) using specific primers (Forward: 5′-GAAGGAGATATA*CATATG*CAGCAAACTGTTTGGGGG-3′, Reverse: 5′-CTTCTTATC*GGATCC*GTTGTTGTTGTT­GTTGTTGTTGTTGTTGTTGCTGCTGCTAATGCATTG­AGCGTAGTA-3′), in which the underlined characters were introduced for the In-fusion™ Advantage PCR cloning kit (Clontech, USA). The* italic* letters indicate the site of the restriction enzymes. The polymerase chain reaction (PCR) product containing CBD with S_3_N_10_ linker was introduced into the pET-28b(+) plasmid (Novagen, USA), previously digested with* Nde*I and* BamH*I. For the isolation of genomic DNA of* E*.* rhusiopathiae*, a DNeasy Blood and Tissue Kit (Qiagen, Germany) was used according to manufacturer's instructions. The surface protective antigen A (SpaA) gene was amplified from* E*.* rhusiopathiae* genomic DNA, by PCR using the primers (Forward: 5′-ACTCCGCCAACTAGCTCT*GGATCC*GATTCGACAGAT­ATTTCTG-3′, Reverse: 5′-GTGGTGGTGGTGGTGGTG­*CTCGAG*TTTTAAACTTCCATCGTTC), in which underlined base pairs were introduced for In-fusion ligation. The* italic* letters indicate the sites of* BamH*I and* Xho*I.

### 2.3. Expression and Purification of CBD-SpaA Protein

The plasmid harboring pKPM-CBD-Lk-SpaA-H6 was transformed into* E. coli* BL21(*DE3*). The transformants were grown in 2YT medium containing 50 *μ*g/ml of kanamycin at 37°C. When an A_600_ of 0.5 was reached, 0.5 mM isopropyl-*β*-thio-D-galactopyranoside (IPTG) was added and cultures were grown for an additional 3h at 37°C. Cells were harvested by centrifugation and pellets were disrupted by sonication for further analysis. Recombinant fusion protein was purified from the supernatant by Ni-NTA agarose (Qiagen, Germany), using an Econo-Pac Chromatography Column (Bio-Rad, USA) according to the manufacturer instructions. The protein concentration was estimated using the BCA protein assay kit (Pierce, USA).

### 2.4. Binding of CBD-SpaA onto Avicel

Binding assays were carried out according to methods previously described, with slight modification [19]. Briefly, Avicel (PH-101, Sigma, < 50 *μ*m) (0.1 mg) was mixed with 500 *μ*g of the CBD-SpaA proteins at 4°C for 2 h. The supernatant was collected after centrifugation and used to determine uncombined protein using a BCA protein assay kit. The amount of protein bound to Avicel was determined from the difference between final and starting values in the supernatant. Nonspecific proteins bound to Avicel were removed by washing with 0.5 M NaCl.

### 2.5. SDS-PAGE and Western Blot Analysis

SDS-PAGE (sodium dodecyl sulfate-polyacrylamide gel electrophoresis) was performed according to the method of Laemmle [20]. For the Western blot analyses, proteins separated by 10% SDS-PAGE gel were transferred to nitrocellulose (NC) membranes. The NC membranes were blocked by phosphate buffered saline (PBS) containing 5% skimmed milk and washed three times with PBST (0.1% Tween 20 in PBS). The membranes were incubated with His-probe monoclonal antibody (Santa Cruz Biotechnology, USA) or mouse-derived antiserum prepared with whole bacteria of* E*.* rhusiopathiae*, for 1 h at room temperature (RT). The specific antigen reacted with IgG alkaline phosphatase (AP) antibody (Sigma, USA) was visualized using an AP conjugated substrate kit (Bio-Rad, USA).

### 2.6. Preparation of Polyclonal Mouse Antiserum

To prepare mouse polyclonal antiserum against* E*.* rhusiopathiae*, a five-week-old specific-pathogen-free (SPF) mouse was injected subcutaneously with formalinized whole cell vaccine of* E*.* rhusiopathiae* twice within a 2-week interval. Then, a blood sample was collected two weeks later and sera antibody titer was measured using enzyme-linked immunosorbent assay (ELISA).

### 2.7. Antigenicity Evaluation

Microtiter assembly strips (Thermo Scientific, Finland) were coated overnight at 4°C with 100 *μ*l per well of immobilized CBD-SpaA on Avicel. Several dilutions (1×10^4^, 5×10^4^, 1×10^5^, and 5×10^5^ Avicel particles) of Avicel coated CBD-SpaA were tested in triplet. The plates were washed three times with PBST and blocked with 5% skimmed milk in PBST for 1h at RT. Antiserum derived from mouse against* E*.* rhusiopathiae* (1:500) was added to the plates and then placed on a rocker platform for 2h at RT. For the detection of immunogenic characteristics, the plates were incubated with a 1:2000 dilution of horseradish peroxidase (HRP) anti-mouse IgG whole antibody (GE Healthcare, UK) for 1 h at RT. Optical density was read at 450 nm using a TECAN Infinity 2000 PRO plate reader (TECAN, Austria).

### 2.8. Confocal Laser Scanning Microscope (CLSM)

Avicel particles (1×10^5^) coated with CBD-SpaA were incubated with antiserum against* E*.* rhusiopathiae* (1:500) for 2h at RT. After washing three times with PBST, the immobilized CBD-SpaA was incubated with Fluorescein- (FITC-) AffiniPure F(ab')2 Fragment Goat Anti-Mouse IgG (H+L) (Jackson ImmunoResearch Lab, USA) for 30 min at 4°C. The plate was washed again, and Avicel particles coated with CBD-SpaA were fixed with 3.7% paraformaldehyde for 10 min at RT, followed by mounting with VECTASHIELD mount medium (Vector Lab., USA). Immunofluorescence was evaluated using an LSM 510 META Laser Scanning Microscope (Carl Zeiss, Germany).

### 2.9. Mouse Immunization and Challenge

Forty SPF mice (5 weeks old) were randomly assigned to 4 groups of eight each and injected subcutaneously with 4 *μ*g of soluble CBD-SpaA, Avicel coated with CBD-SpaA, ERT2T-A containing whole bacterin of* E*.* rhusiopathiae *serovar 15 (positive control), and PBS (negative control) emulsified with an oil-based adjuvant, respectively. Two weeks after injection, all groups were challenged subcutaneously with 1×10^7^ CFU of* E*.* rhusiopathiae *(serovar 15). Mouse mortality was monitored daily for the following ten days.

## 3. Results

### 3.1. Expression of the CBD-SpaA Fusion Protein in E. coli

**Figure 1:**
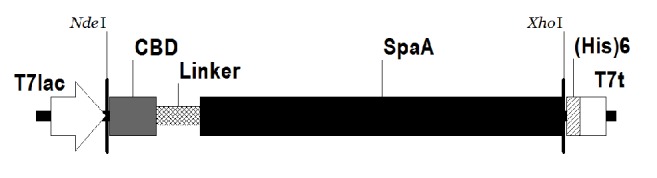
A schematic representation of the expression vector for CBD-SpaA-H6 fusion protein. SpaA, surface protective antigen A (SpaA) from* E. rhusiopathiae*; CBD, the cellulose-binding domain of* T. harzianum* endoglucanase II; Linker, S_3_N_10_ peptide; (His)6, 6x histidine tag sequence; T7lac, T7 promoter sequence; T7t, T7 terminator sequence.

**Figure 2:**
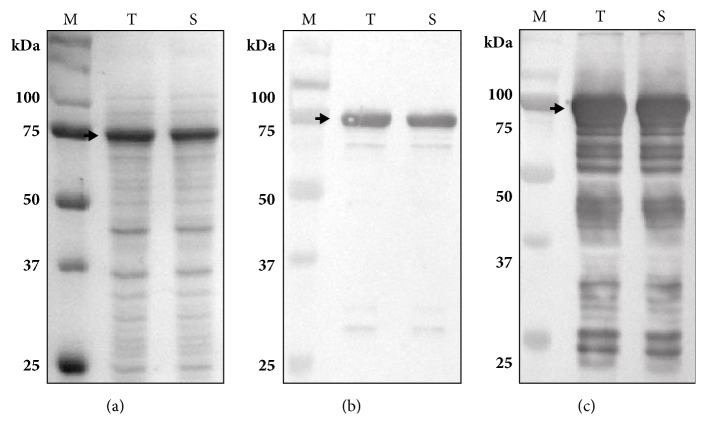
SDS-PAGE and Western blot analyses of the CBD-SpaA-H6 expressed in* E. coli* BL21(DE3)/pKPM-CBD-SpaA-H6: (a) SDS-PAGE; (b) Western blot with anti-histidine antibody; (c) anti-serum derived from mouse inoculated with whole* E. rhusiopathiae*. Lane M: standard molecular weight marker, T: total cell lysates, and S: soluble fraction proteins. The arrows indicate the expressed CBD-SpaA.

As illustrated in Figure 1, pKPM-CBD-Lk-SpaA-H6 was constructed for the expression of SpaA fused to CBD. In the construct, SpaA was fused to CBD from* T. harzianum *endoglucanase II via an artificial S_3_N_10_ peptide known to completely resist* E. coli* endopeptidase at its N-terminus and six histidines at its C-terminus. The fusion protein (CBD-SpaA) was expressed in* E. coli* BL21(*DE3*) harboring pKPM-CBD-Lk-SpaA-H6. The results of the SDS-PAGE and Western blot (Figure 2) indicated that the CBD-SpaA was successfully expressed at the expected molecular weight (77.6 kDa) without being degraded by proteolysis and that the CBD-SpaA was overexpressed at a high level (35%) with respect to the percentage of total cell protein. Moreover, most of the expressed CBD-SpaA were in soluble form, compared to our previous work in which extreme reduction of the solubility of* E. coli*-derived glutamate decarboxylase occurred after fusion with CBD [21].

### 3.2. Coating of Avicel with CBD-SpaA Protein

**Figure 3:**
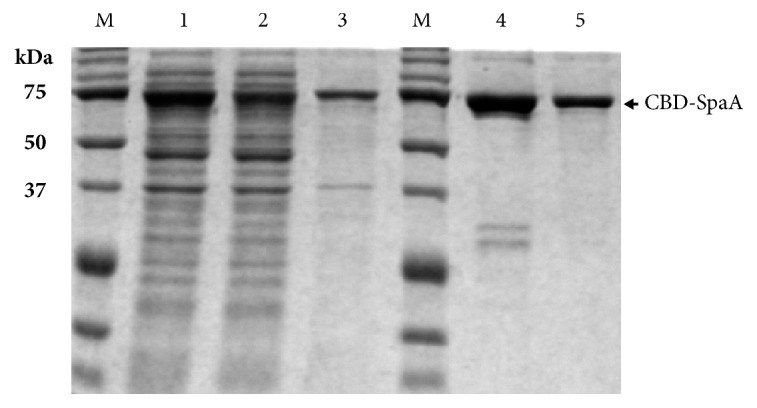
Coating of Avicel with CBD-SpaA protein. Lanes 1-3: crude CBD-SpaA and Lanes 4 and 5: purified CBD-SpaA. Lane M: stand molecular weight marker, Lanes 1 and 4: proteins before binding to Avicel, Lane 2: proteins not bound to Avicel, and Lanes 3 and 5: proteins bound on Avicel.

The CBD-SpaA proteins from crude cell lysates were purified through a Ni-NTA column with the elution of an imidazole (250 mM). The elution fraction contained CBD-SpaA with a purity of 85.1%. Both the purified CBD-SpaA and crude cell lysates were bound to microcrystalline cellulose Avicel PH-101 and the concentration of CBD-SpaA bound to Avicel was determined by stripping the protein from the beads by boiling. Avicel displayed a binding capacity of 3.11±0.015 mg_CBD-SpaA_/g_Avicel_ (purity: 94%) for the purified CBD-SpaA and 1.92±0.001 mg_CBD-SpaA_/g_Avicel_ (purity: 81%) for crude cell lysates (Figure 3).

### 3.3. Antigenicity of CBD-SpaA Bound to Avicel

**Figure 4:**
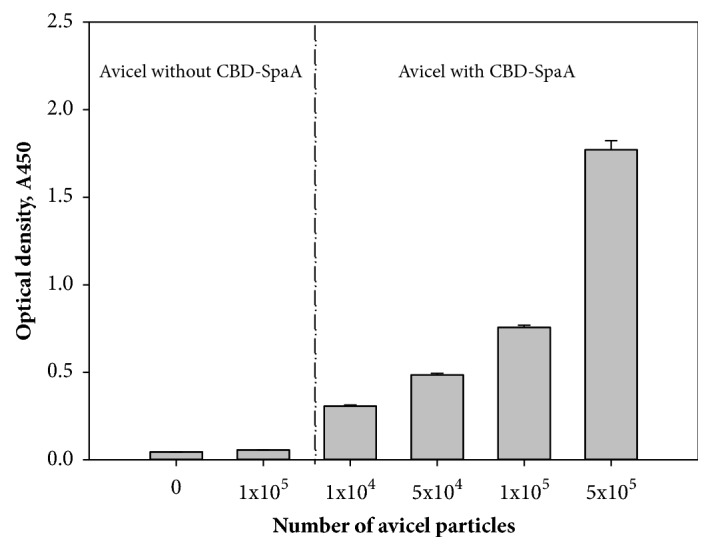
ELISA assay of Avicel coated with CBD-SpaA (*n=3*).

**Figure 5:**
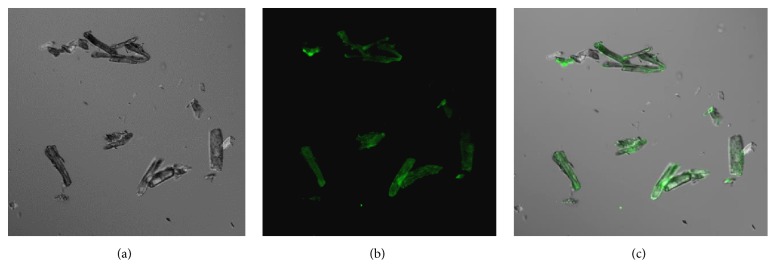
CLSM images for CBD-SpaA-H6 immobilized on Avicel. Purified CBD-SpaA protein was bound to Avicel and incubated with anti-histidine antibody followed by goat anti-mouse IgG-FITC. The confocal microscope image indicates that CBD-SpaA protein had a high binding affinity to Avicel. (a) Differential interference contrast images; (b) FITC fluorescence; (c) merging of the two split images.

The antigenicity of CBD-SpaA bound to Avicel was evaluated by indirect ELISA assay. As shown in Figure 4, the increased absorbance values were detected in proportion to the number of Avicel particles coated with CBD-SpaA, whereas the Avicel without CBD-SpaA as a negative control showed a value as low as the PBS. Confocal laser scanning microscope (CLSM) was used for visualization of antigenic properties of the Avicel coated with CBD-SpaA (Figure 5). Purified fusion protein was bound to Avicel and incubated with anti-serum of whole* E. rhusiopathiae*, followed by goat-mouse IgG-FITC. CLSM images demonstrated that green fluorescence dispersed almost equally on the surface of the Avicel coated with CBD-SpaA as shown in Figure 5.

### 3.4. Protective Immunity in Immunized Mice

**Figure 6:**
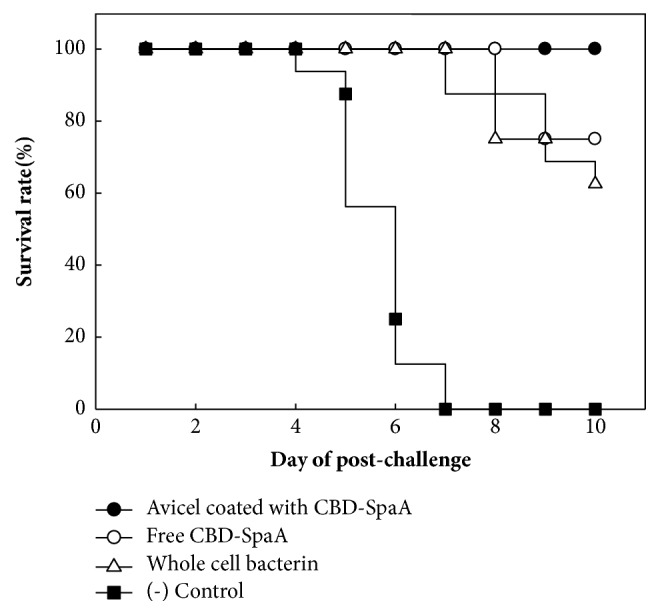
Cumulative mortality of immunized mice after challenge. Mice were vaccinated with free CBD-SpaA, Avicel coated with CBD-SpaA, and ERT2T-A containing whole cell bacterin as a positive control and PBS as a negative control. After 14 days, all the mice were challenged with highly virulent* E. rhusiopathiae* and survival was monitored for ten days.

To examine whether protection could be induced without side effects by immunization with the Avicel coated with CBD-SpaA, we injected each protein (free CBD-SpaA and Avicel coated with CBD-SpaA, ERT2T-1 containing whole cell bacterin (positive control) from CVAVC in the Republic of Korea, and PBS (negative control)) into mice and challenged the mice with* E. rhusiopathiae*. All of the negative control mice died within seven days after challenge. Compared with negative control group, 5 of 8 (p < 0.0001, by Fisher's exact test) mice in the ERT2T-1 immunized group, and 6 of 8 (p < 0.0001, by Fisher's exact test) mice in the free CBD-SpaA immunized group, survived. In the Avicel coated with CBD-SpaA, the immunized group of mice showed 100% (p < 0.0001, by Fisher's exact test) immune-protection against challenge with* E. rhusiopathiae *(Figure 6). However that group did not show significant difference with the free CBD-SpaA immunized group (p = 0.467, by Fisher's exact test). These results demonstrated that the microcrystalline cellulose can be used as a delivery carrier of recombinant protein.

## 4. Discussion

Antigen subunits or synthetic peptides are considered as a promising alternative for viral vaccines. They have been also considered safer vaccine systems than killed/inactivated or live-attenuated whole cells/viruses. Recombinant DNA technology has made the development of subunit vaccines more efficient, because the production and purification procedure can be carefully designed to obtain high yields of a well-defined product [22]. However, some studies have shown that soluble immunogens rarely induce high titers of antibodies without the use of strong adjuvants [23, 24]. To overcome the typical low immunogenicity of protein-based vaccines, and to address the need for effective vaccines and efficient delivery systems, researchers have moved in the direction of molecular biotechnology [25]. Recently, advances in recombinant biotechnology have led to the development of genetically engineered polymers with exact order and accuracy of amino acid residues. Recombinant protein-based polymers such as elastin-like polymers (ELPs), silk-like polymers (SLPs), and silk-elastin-like protein polymers (SELPs) have been reported to bring controlled release, longer circulating therapeutics, and tissue-specific treatment options [26]. Nanoscale structures such as gold nanoparticles and virus-like particles have also recently peaked interests for drug delivery as they offer manifest benefits [27, 28].

In our study, Avicel was selected as immunosorbent for purification and delivery of recombinant protein subunit vaccine, because it is highly inert, inexpensive, and safe biomaterial. This should protect the protein subunit vaccine from degradation in harsh conditions (extreme pH or temperature). As shown in Figure 4, immune response increased with increasing Avicel particle concentration, confirming its suitability as a better immunosorbent for vaccine systems. The higher immunity achieved with increased Avicel concentration could be due to selective coating of SpaA by Avicel.

Our findings clearly demonstrate that Avicel is specific for the CBD-SpaA with good binding capacity for vaccine delivery. The binding capacity was visualized using CLSM imaging as shown in Figure 5. It has been well known that the Spa proteins are potent protective antigens against* E. rhusiopathiae* infection [29]. Recent reports show that SpaA is the major Spa-type of serotypes 1a, 1b, and 2 [30] which are most commonly implicated in swine erysipelas. The protective domain of SpaA lies between amino acids 29-414 [30]. This particular amino acid sequence itself can induce highly protective antibodies against* E. rhusiopathiae* infection [15]. Being a well delivery agent and immune sorbent, Avicel further enhanced its potency as we found in our experiments.

## 5. Conclusions 

In this study, we developed a vaccine using microcrystalline cellulose, Avicel PH-101 to deliver recombinant protein-based antigen against erysipelas. The recombinant protein, CBD-SpaA, was expressed in* E. coli* and bound to Avicel PH-101. Our data perceptibly showed that a 100% survival rate could be achieved using the Avicel coated with antigen in* E. rhusiopathiae*-challenged mice. Thus, the in vitro immunogenicity test has been validated by the in vivo challenge experiment.

In our newly developed vaccine system, protein purification is unnecessary. This cuts down production costs and enables a cost-effective, recombinant protein vaccine system. Specifically, our delivery system using Avicel coated with CBD-SpaA could provide an appealing vaccination strategy against erysipelas.

## Data Availability

The data used to support the findings of this study are available from the corresponding author upon request.
